# The microbial community structure in industrial biogas plants influences the degradation rate of straw and cellulose in batch tests

**DOI:** 10.1186/s13068-016-0543-9

**Published:** 2016-06-18

**Authors:** Li Sun, Tong Liu, Bettina Müller, Anna Schnürer

**Affiliations:** Department of Microbiology, Swedish University of Agricultural Science, Uppsala BioCenter, P.O. Box 7025, 750 07 Uppsala, Sweden; Department of Chemistry, Biotechnology and Food Science, Norwegian University of Life Science, 1432 Ås, Norway

**Keywords:** Biogas, Cellulose, Community composition, Glycoside hydrolases, *cel48*, *cel5*, Terminal restriction fragment length polymorphism (T-RFLP), Next generation amplicon sequencing

## Abstract

**Background:**

Materials rich in lignocellulose, such as straw, are abundant, cheap and highly interesting for biogas production. However, the complex structure of lignocellulose is difficult for microbial cellulolytic enzymes to access, limiting degradation. The rate of degradation depends on the activity of members of the microbial community, but the knowledge of this community in the biogas process is rather limited. This study, therefore, investigated the degradation rate of cellulose and straw in batch cultivation test initiated with inoculums from four co-digestion biogas plants (CD) and six wastewater treatment plants (WWTP). The results were correlated to the bacterial community by 454-pyrosequencing targeting 16S rRNA gene and by T-RFLP analysis targeting genes of glycoside hydrolase families 5 (*cel5*) and 48 (*cel48*), combined with construction of clone libraries

**Results:**

UniFrac principal coordinate analysis of 16S rRNA gene amplicons revealed a clustering of WWTPs, while the CDs were more separated from each other. Bacteroidetes and Firmicutes dominated the community with a comparably higher abundance of the latter in the processes operating at high ammonia levels. Sequences obtained from the *cel5* and *cel 48* clone libraries were also mainly related to the phyla Firmicutes and Bacteroidetes and here ammonia was a parameter with a strong impact on the *cel5* community. The results from the batch cultivation showed similar degradation pattern for eight of the biogas plants, while two characterised by high ammonia level and low bacterial diversity, showed a clear lower degradation rate. Interestingly, two T-RFs from the *cel5* community were positively correlated to high degradation rates of both straw and cellulose. One of the respective partial *cel5* sequences shared 100 % identity to *Clostridium cellulolyticum.*

**Conclusion:**

The degradation rate of cellulose and straw varied in the batch tests dependent on the origin of the inoculum and was negatively correlated with the ammonia level. The cellulose-degrading community, targeted by analysis of the glycoside hydrolase families 5 (*cel5*) and 48 (*cel48*), showed a dominance of bacteria belonging the Firmicutes and Bacteriodetes, and a positive correlation was found between the cellulose degradation rate of wheat straw with the level of *C. cellulolyticum*.

**Electronic supplementary material:**

The online version of this article (doi:10.1186/s13068-016-0543-9) contains supplementary material, which is available to authorized users.

## Background

Anaerobic digestion (AD) transforms biodegradable organic materials into a renewable energy resource, biogas, which can be used for production of vehicle fuel and/or for combined heat and electricity generation [1]. Furthermore, the residue after digestion is rich in nutrients and can be used as a fertiliser in crop production [2]. In a number of studies, production of biogas has been shown to offer significant advantages over other forms of bioenergy production and it has been rated one of the most energy-efficient and environmentally beneficial technologies for bioenergy production [3, 4]. Biogas can be produced from many different types of materials, including various types of waste streams from the food and feed industry, sludge from wastewater treatment plants, plant residues and manure from agriculture and energy crops [5, 6]. Plant-based biomass is very interesting in this regard, with lignocellulosic residues being the most promising material as these do not compete directly with food and feed production [7]. They include residues of agricultural plants, e.g. stalks, straw, husks, cobs, etc. The total amount of lignocellulosic residues accumulated annually in the world is estimated to be at least 10 billion tons [8].

Unfortunately, biogas production from lignocellulose-rich materials poses some challenges, as the complex structure, consisting of cellulose, hemicellulose and lignin cross-linked in a matrix structure, is very resistant to microbial degradation [9–13]. These difficulties can be overcome to some degree by a pre-treatment that breaks up the complex structure and makes the material more accessible for degradation, but the degradation rate and biogas yields are still typically rather low [7]. Moreover, many pre-treatments are energy- and cost-intensive, limiting large-scale application. Alternative less energy-consuming approaches to improve the degradation of lignocellulosic materials include management of the biogas process to ensure growth of microorganisms with efficient degradation capabilities, for example by adjustment of the solid retention time, using a two-stage approach or by a co-digestion approach as reviewed by Sawatdeenarunat and co-workers [7]. Bioaugmentation with efficient cellulose-degrading bacteria and addition of enzymes have also been suggested as promising methods to increase methane production from lignocellulosic materials [14, 15].

The microbial degradation of organic materials for biogas production requires at least four steps: hydrolysis, fermentation, acetogenesis and methanogenesis [16, 17]. In the first step, various hydrolytic microorganisms degrade complex organic polymers to monomers such as amino acids and sugars. In this step, microbes responsible for cellulose degradation use either free extracellular or cell-anchored enzyme complexes including cellulosomes, the latter more commonly found in anaerobic environments [10, 18]. A recent survey of around 1500 complete bacterial genomes revealed that ~38 % of the sequenced bacterial genomes encoded at least one cellulase gene, with a small fraction containing more than three cellulases, a prerequisite for effective degradation of natural cellulose [19]. The genes necessary for degradation of cellulose have been found in bacteria belonging to several different phyla: Actinobacteria, Fimicutes, Bacteroidetes, Thermotogae, Choloroflexi and Proteobacteria [19–21]. Cellulose-degrading bacterial communities specifically in biogas processes have been investigated by various methods. These include cultivation [22–27] and different molecular techniques targeting bacterial groups involved specifically in hydrolysis/acetogenesis [28] and functional genes, i.e. the glycoside hydrolase [29], or targeting the overall bacterial community [30–34]. The majority of cultivated cellulose degraders isolated from different anaerobic environments mainly belong to the order Clostridiales [10, 20, 21]. Bacteria from this order have also been shown to dominate in various AD processes operating with various lignocellulosic materials, such as wheat straw and cattle manure [31], maize, green rye and chicken manure [35], and maize straw and hay [36]. Clostridiales is also suggested to be of importance, specifically in the hydrolysis step, based on results obtained using a metagenomic approach [30]. In addition to Clostridiales, bacteria belonging to the order Bacteroidales have been suggested to be involved in the degradation of lignocellulose materials, such as straw and hay, in biogas processes [31, 36].

A range of studies have examined different technical/chemical/thermal methods for enhanced degradation of lignocellulosic materials and lately knowledge regarding the microbiological mechanisms, and organisms involved in the degradation of lignocellulose have also increased. However, the correlation between the structure of the cellulose-degrading community in a biogas process and efficient degradation is still unclear and requires further research. The aim of this study was, therefore, to search for correlations between the degradation rate of cellulose and straw and the bacterial community structure, including potentially cellulose-degrading bacteria. An additional aim was to obtain further information about the cellulose-degrading bacterial population in different AD processes. The degradation of straw and cellulose was analysed using a batch culture approach and with inoculums from ten different Swedish biogas plants, operating with different substrates and with different operating parameters. These inoculum samples were analysed both before and after the batch cultivation to determine the composition of potential cellulose-degrading bacteria by targeting the glycoside hydrolase genes of family 5 and family 48 glycoside hydrolases. The total bacterial community in the different biogas plants was also analysed, using next generation amplicon sequencing targeting the 16S rRNA gene.

## Results and discussion

### Characterisation of inoculum

The inoculum samples investigated in this study originated from four co-digestion plants processing various combinations of substrates (CD01–04) and six wastewater treatment plants processing mixed sludge (WWTP01–06). In general, the co-digesters had a longer hydrolytic retention time (HRT) and higher levels of VFA and total ammonium-nitrogen than the WWTP (Table 1). However, one co-digestion plant (CD03) was more similar to the WWTP in this respect. Moreover, the free ammonia level in the three plants CD01, 02 and 04 was considerably higher (>0.218 g L^−1^) than in the other plants (<0.063 g L^−1^).

**Table 1:** Operating data for the 10 industrial-scale biogas plants investigated in this study

Digester code	TS^a^ (%)	VS^b^ (%)	HRT^c^ (day)	TM^d^ (°C)	Tot N^e^ (g L^−1^ ww)	pH	VFA^f^ (g L^−1^)	OLR^g^ (vs g L^−1^ day^−1^)	TAN^h^ (g L^−1^ ww)	Ammonia^i^ (g L^−1^ ww)	Major substrate
CD01	5.7	3.5	45	38	8.7	7.8	1.3	3.0	4.6	0.365	SSMOW^j^, slaughterhouse waste
CD02	4.8	3.8	55	38	9.1	7.8	0.8	2.9	5.1	0.408	Thin stillage
CD03	2.6	2.0	30	37	2.6	7.3	<0.1	3.0	0.9	0.022	SSMOW
CD04	7.1	1.3	70	38	6.2	7.7	3.0	3.0	3.5	0.218	Grass, wheat-based stillage
WWTP01	2.7	1.9	17	38	2.5	7.3	<0.1	2.4	1.4	0.036	Mixed sludge
WWTP02	3.5	2.5	23	37	2.9	7.5	0.2	3.1	1.6	0.063	Mixed sludge
WWTP03	2.4	1.9	18	38	1.8	7.3	0.1	2.0	0.9	0.022	Mixed sludge
WWTP04	3.4	2.4	30	37	2.9	7.5	0.3	1.6	1.5	0.058	Mixed sludge
WWTP05	2.5	2.1	22	37	2.1	7.3	0.1	2.8	1.1	0.027	Mixed sludge
WWTP06	2.1	5.5	26	34	1.8	7.3	<0.1	1.1	1.1	0.023	Mixed sludge

*CD 01–04* co-digestion plants, *WWTP 01–06* wastewater treatment plants

^a^Total solids

^b^Volatile solids

^c^Hydraulic retention time

^d^Temperature

^e^Total nitrogen

^f^Volatile fatty acids

^g^Organic loading rate

^h^Total ammonium nitrogen

^i^Free ammonia, calculated according to Hansen et al. [106]

^j^Source-separated municipal organic waste

### BMP analysis

The final methane potential achieved with inoculum from the different industrial-scale biogas plants varied from 307 ± 54 to 376 ± 8 N mL CH_4_ g VS^−1^ for cellulose and from 233 ± 38 to 316 ± 37 N mL CH4 g VS^−1^ for straw (Table 2). The degradation rate and the time needed to reach the final potential also varied, with a clearly lower degradation rate in the tests initiated with inoculum from CD01 to 02. The time needed to reach 50 % of the final potential with these two inoculum samples was 18–47 days and 28–45 days for cellulose and straw, respectively, while for the other inoculum samples the corresponding values were 5–8 and 8–19, respectively. The methane potential values obtained for cellulose and straw were in line with those reported in previous studies, illustrating that the inoculum samples evaluated in this study came from fully functional biogas plants [37–39]. Some of the tests showed large standard deviation, most likely due to the non-homogeneous character of some inoculum samples making it difficult to divide them evenly between the triplicate bottles. It is also worth noting that the inoculum:substrate ratio inadvertently differed slightly between the different inoculum samples, but was still within the range suggested as optimal for a BMP test, i.e. 2–4. However, this difference could potentially have had an impact on the degradation rate and possibly also on the final potential in the test. The ratio for the inoculum from WWTP04–06 was adjusted to 3.8, 3.2 and 2.4, respectively, while the rest of the tests started with a ratio of 2. Even so, the degradation rate obtained in the tests of CD01–02 was still lower than for CD03–04 and WWTP01–03, despite all these sharing the same ratio.

**Table 2:** Methane potential and time for degradation of straw and cellulose obtained in biochemical methane potential tests using inoculum from different biogas plants co-digesting different substrates (CD) or sludge from wastewater treatment plants (WWTP)

Inoculum	Cellulose				Straw			
	Days to reach % of the final potential			Final potential	Days to reach % of the final potential			Final potential
	100 %	80 %	50 %		100 %	80 %	50 %	
CD01	41	24	18	319 ± 24	110	56	28	258 ± 47
CD02	110	71	47	307 ± 54	75	62	45	233 ± 38
CD03	12	6	5	347 ± 15	60	23	8	316 ± 37
CD04	20	10	8	348 ± 24	26	10	8	274 ± 17
WWTP01	57	15	7	350 ± 7	110	44	19	290 ± 9
WWTP02	36	13	7	314 ± 34	57	24	14	240 ± 38
WWTP03	27	11	6	322 ± 7	75	25	13	310 ± 76
WWTP04	29	15	8	325 ± 8	60	20	11	277 ± 11
WWTP05	45	9	6	376 ± 8	59	30	10	281 ± 32
WWTP06	49	13	8	324 ± 13	135	39	18	296 ± 8

### Bacterial communities

#### Diversity indices

**Fig. 1:**
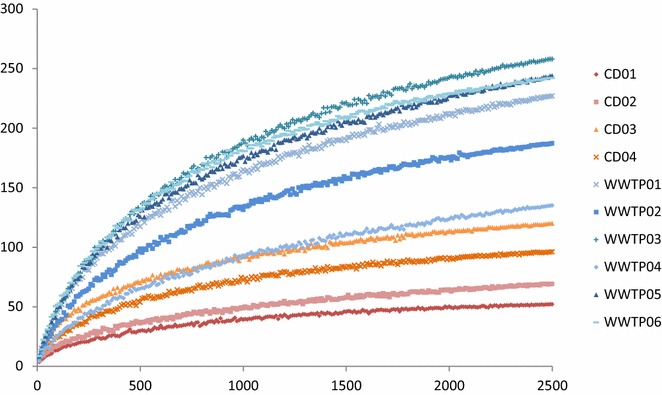
Rarefaction analysis of bacterial communities in 10 industrial-scale biogas plants. *CD 01–04* co-digestion plants, *WWTP 01–06* wastewater treatment plants

Analysis of the bacterial communities in the ten industrial-scale biogas plants by 454 pyrosequencing resulted in 36,523 sequences after quality trim and chimera check, with a range from 2573 to 4915 sequences per sample. Unique barcode was assigned to each replicate of one sample. The triplicate sequencing analysis was evaluated with unweighted UniFrac principal coordinate analysis (PCoA), where no outlier was observed (data not shown). The triplicates were then pooled in silico and randomly subsampled according to the sample having the lowest number of sequences (2500 sequences per sample). The number of OTUs per sample ranged from 52 to 258, with a comparatively lower value for the CD plants (Table 3). The rarefaction curve revealed the same general trend, i.e. a lower number of OTUs in the co-digestion plants compared with the WWTP (Table 3; Fig. 1). Based on the observed species and the Chao1 index, the sequencing covered 64.7–89.7 % of the total bacterial community. The three co-digestion plants CD01, 02 and 04 had low diversity and evenness of the bacterial community, as indicated by low values of species richness, Shannon and Simpson index. However, WWTP04 had similarly low values in this regard (Table 3).

**Table 3:** Summary of observed OTUs, Chao1, Shannon and Simpson index in 10 industrial-scale biogas plants

Sample	Chao 1	OTUs	Shannon	Simpson
CD01	58	52	1.937	0.456
CD02	94	69	3.111	0.763
CD03	147	120	5.042	0.947
CD04	109	96	3.619	0.767
WWTP01	294	227	5.851	0.956
WWTP02	215	187	4.737	0.861
WWTP03	304	258	5.823	0.930
WWTP04	209	135	3.088	0.609
WWTP05	354	244	5.899	0.955
WWTP06	280	242	5.857	0.930

*CD 01-04* co-digestion plants, *WWTP 01-06* wastewater treatment plants

The low diversity in CD01, 02 and 03 correlated with the comparably higher level of ammonia in these digesters (two-sample *t* test and nonparametric Monte Carlo permutations, *n* = 999, *P* < 0.01). In biogas plants processing protein-rich materials, the ammonia released during the AD process is known to have a strong impact on both the community structure and the diversity, most probably because of the inhibitory effects of ammonia [40–42]. High evenness and richness have been suggested to be associated with good conversion efficiency of fatty acids to methane [43]. High initial evenness has also been shown to be important for preserving functional stability in microbial communities in general [44]. In the present study, CD01, 02, 04 and WWTP04 had lower evenness and diversity than the other digesters, implying that these biogas plants might have a comparatively lower potential to reach high degradation efficiencies. Indeed in the BMP tests started with inoculum from CD01 to CD02 a comparably lower methane production rates was seen for both substrates (Table 2). However, both CD04 and WWTP04 showed higher degradation rates, similar to the rates obtained with inoculums having a higher bacterial diversity (CD03, WWTP01–3 and WWTP05–6).

#### Phylogenetic analysis across samples

**Fig. 2:**
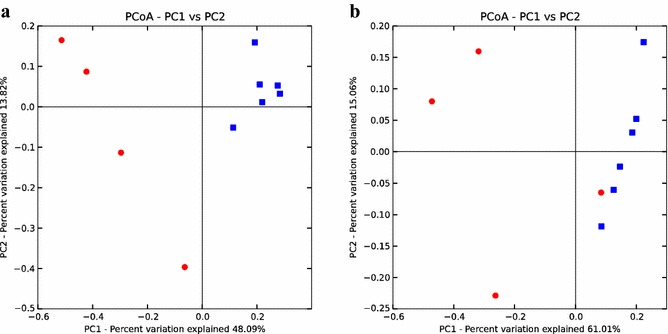
Phylogenetic distance between samples as determined by **a** unweighted and **b** weighted UniFrac principal coordinate analysis (PCoA) (*red* co-digestion plants, *blue* wastewater treatment plants)

**Fig. 3:**
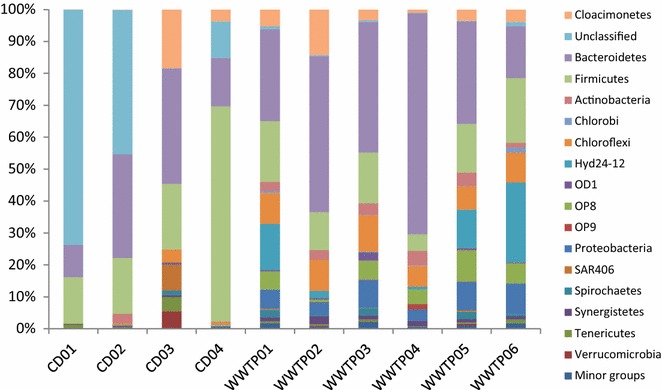
Relative abundance of bacterial 16S rRNA gene at phylum level in 10 industrial-scale biogas plants. *CD 01–04* co-digestion plants, *WWTP 01–06* wastewater treatment plants

The phylogenetic composition, as determined by PCoA of unweighted UniFrac matrices (Fig. 2a), clearly revealed a clustering of the WWTPs, which suggests close phylogenetic distance within these different plants, while the CDs were more separated from each other and from the WWTPs. Considering the relative abundance, as revealed by the weighted UniFrac matrices, the plant CD03 was more closely related to the WWTPs, while the other three CDs plants were still distantly separated from each other and from the WWTPs (Fig. 2b). This confirms findings by Sundberg et al. [45] of two separate clusters distinguishing co-digestion plants and plants processing sewage sludge. In general, Firmicutes and Bacteroidetes were identified as predominant phyla in the industrial-scale biogas plants studied here, but other phyla were also present (Fig. 3). For example, the phyla Chloroflexi, Proteobacteria and OP8 were more associated with plants processing sewage sludge (Welch’s *t*-test, *P* < 0.01), while the co-digesting CD01 and 02 biogas plants contained a large fraction of sequences belonging to a unclassified cluster at phylum level. Bacteroidetes and Firmicutes are the two dominant phyla commonly found in various AD processes [21, 28, 31, 35, 36, 46, 47].

The phylum Firmicutes was detected in all biogas plants, but at different relative abundance, from 5.2 to 67 % (Fig. 3). Within this phylum, the class Clostridia was dominant, with relative abundance of 4.4–64 %. Other classes were also present but at lower levels, such as Bacilli (<2.7 %) and Erysipelotrichi (<0.4 %) (Additional file 1: Figure S1). The order Clostridiales (3.8–56.3 %) was dominant within the clostridia, while Thermoanaerobacterales (<0.6 %) and unclassified orders such as MBA08 and SHA-98 comprised the rest of this class (Additional file 1: Figure S2). In anaerobic environments, Clostridiales has been reported as the main cellulose degrader [10]. This order has frequently been recovered from various digesters operating with mono- and co-digestion of lignocellulosic materials [31, 32, 46, 48–50]. However, the class Clostridia also contains proteolytic members and replacement of cellulolytic clostridia with proteolytic members has been observed when using protein-rich material as substrate with an inoculum originating from a biogas plant processing pig slurry and maize silage [51, 52]. A genus *Caldicoprobacter* (OTU 481, Additional file 1: Figures S4, S5) within Clostridiales was found at a higher relative abundance (0.68 %) in CD01, 02 and 04 compared to the rest samples (<0.04 %). This genus contains several xylanolytic bacteria [53–55]. The order MBA08 was detected in four samples in the present study CD01 (2.8 %), CD02 (1.5 %), CD04 (6.8 %) and WWTP06 (0.4 %) (Additional file 1: Figures S2, S5). This cluster was first identified in a thermophilic laboratory-scale digester [56] and later also in other thermophilic digesters [31, 57]. The presence of representatives from this cluster in the mesophilic biogas plants included in this study suggests that MBA08 contains organisms growing at wide range of temperatures. The order SHA-98 was observed at comparably higher levels in the high ammonia digesters CD01 and CD02, with relative abundance of 5.5 and 8.8 %, respectively. The levels in the rest of the digesters were 0.3–3.2 %. This order was represented by two dominant OTUs, OTU11 representing the main OTU in the high ammonia digesters CD01/02/04 (up to 7.9 %) and OTU 35, comparably more abundant in all other digesters (up to 2.6 %) (Additional file 1: Figure S5). In line with previous findings by Sundberg and co-workers [45], the family Clostridiaceae had higher relative abundance in WWTPs (2.2–5.4 %) than in CDs (0.2–0.9 %) (Welch’s *t* test, *P* < 0.01) (Additional file 1: Figure S3). Notably, the genus *Gallicola*, classified to the proposed family Tissierellaceae [58], was present at high relative abundance in plant CD04 (46.2 %) (Additional file 1: Figure S5). This family was not detected in any other biogas plant except WWTP06 (0.1 %) (Additional file 1: Figure S4). The first representative of the genus *Gallicola* was isolated from chicken manure and it has been shown to grow on purines, such as uric acid, xanthine, 6,8-dihydroxypurine, guanine and hypoxanthine [59].

The phylum Bacteroidetes had a relative abundance of 10.1–69.2 % (Fig. 3). Within this phylum, the order Bacteroidales (class Bacteroidia) was dominant in all samples (Additional file 1: Figure S2, S3). Members of the Bacteroidetes are able to degrade various polysaccharides [60, 61]. In a study with batch fermentation of straw and hay, the relative abundance of Bacteroidetes was higher at the end than at the beginning of the batch cultivation, indicating the importance of this phylum for degradation of lignocellulose [36]. An increase in relative abundance of this phylum has also been observed in response to straw addition in a laboratory-scale digester initially operating with cattle manure [31]. However, the Bacteroidetes have been shown to decrease in abundance at high levels of ammonia [41, 42, 51, 62]. In line with this, the lowest relative abundance of this phylum was seen for CD01 and CD04, both with relatively high ammonia levels (Fig. 3). However, CD02 also had a high ammonia level but had a similar relative abundance of Bacteroidetes as the low ammonia digesters. At family level, the Bacteroidaceae (up to 2.3 %), Marinilabiaceae (up to 0.2 %), Porphyromonadaceae (up to 9.2 %), Rikenellaceae (up to 0.7 %) and a few unclassified families were present (Additional file 1: Figure S3). One unclassified family, SB-1, was also observed, with higher abundance in CD03 (12.6 %) and WWTP01-06 (4.0–65.3 %) than in CD01, 02 and 04 (<0.2 %), possibly suggesting sensitivity to high ammonia levels (Table 1; Additional file 1: Figure S5). In addition, the Porphyromonadaceae were observed at relative abundance of 9.8 % in CD02 and 8.0 % in CD03, while other orders represented less than 2.0 %.

The candidate phylum Cloacimonetes (formerly WWE1) was observed at levels of less than 0.1 % in plants CD01 and CD02, but ranged from 1.0 to 14.2 % in the other eight biogas plants (Fig. 3; Additional file 1: Figure S5). Representatives of this phylum were first discovered in AD plants processing sewage sludge [63], but have since been found in a full-scale plant fed energy crop (mainly maize silage) [32] and a laboratory-scale digester fed cattle manure as the sole substrate or co-digested with wheat straw [31]. A study using stable isotopes suggested that members of this phylum are engaged in either cellulose hydrolysis or uptake of cellulose fermentation products [64]. The uncultured cluster at phylum level SAR406 was observed in CD03 with a relative abundance of 8.2 %, while the level in CD01-02 and in all WWTP was less than 1 % (Fig. 3; Additional file 1: Figure S5). The candidate name Marinimicrobia has been proposed for this cluster [65] and preliminary genome analysis suggests that members within this phylum are proteolytic and amino acid degraders [64, 66]. The phylum Proteobacteria was present at a level of 3.4–9.6 % in the WWTP, but less than 0.3 % in the CDs (Fig. 3; Additional file 1: Figure S5). This phylum has previously been found in various digesters processing sewage sludge [45, 67–69], but recently also in mono-digestion of fodder beet silage [46] and in a process co-digesting food waste with Chinese silvergrass [70]. Representatives of the uncultured phylum Hyd24-12 corresponded to 12–25 % in WWTP 01, 05 and 06, but were much less abundant in the other biogas plants (<2.3 %). This uncultured cluster has been found in other methanogenic digesters [71, 72], but its function in the methanogenic environment is still unknown [73]. The phylum Chloroflexi was represented with relative abundance of 6.5–11.5 % and 0.1–3.9 % in the WWTPs and CDs, respectively, and was dominated by an uncultured genus T78 (5.4–9.2 % in WWTPs and 0.1–3.0 % in CDs) (Fig. 3; Additional file 1: Figure S5). This phylum has been previously found in anaerobic digesters processing sewage sludge [74] and in co-digestion of whey permeate and cow manure [75], and members have been suggested to be carbohydrate utilisers [76, 77]. In addition, an uncultured cluster was found at high levels in CD01, 02 and 04, with a relative abundance of 73.7, 45.2 and 11.4 %, respectively (Fig. 3; Additional file 1: Figure S5). This large cluster was represented by one single OTU and had 84 % similarity based on 16S rRNA gene with an uncultured *Clostridium* (OTU1). The dominance of the community by such a large fraction represented by a single OTU is somewhat surprising and, to our knowledge, has not been reported previously for biogas digesters. Considering the feedstock for these biogas plants, this OTU might represent a protein-fermenting bacterium enriched by the protein-rich feedstock. Alternatively, this bacterium has a selective advantage at the high levels of the free ammonia in these digesters (0.218–0.408 g L^−1^). Ammonia has in previous studies been shown to have a strong selective pressure on the microbial community [41, 42].

### T-RFLP

#### *cel5*

**Fig. 4:**
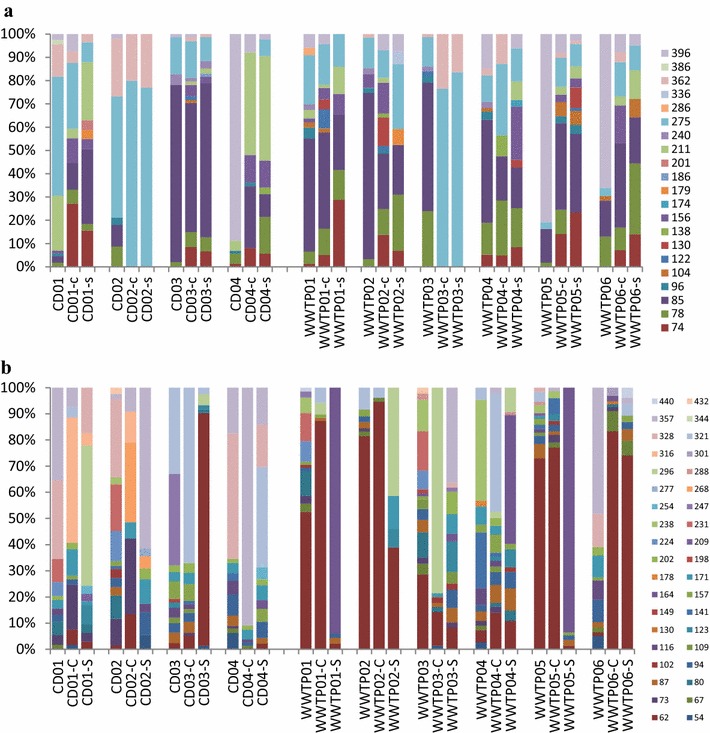
T-RFLP profile representing the community of **a** glycoside hydrolase gene family 5 (*cel5*) and **b** glycoside hydrolase gene family 48 (*cel48*) in 10 industrial-scale biogas plants: CD01–04/c/s and WWTP 01–06/c/s refer to inoculum analysed at the starting point of a batch cultivation and at the end point using cellulose (-c) and straw (-s) as substrate, respectively

The cellulolytic community structures were investigated using T-RFLP combined with clone library analysis (Fig. 4a). Analysis of the inoculum samples revealed that T-RF 275 bp was present in all digesters except CD04, with a higher relative abundance in CD01–02 (51.1–52.1 %) than in the other plants (<21.1 %). T-RF 362 bp was also present at higher levels in CD01–02 (14.0–24.8 %) than in the other samples, with detectable levels only in samples WWTP02–04 (1.3–2.9 %). T-RF 85 bp had the highest relative abundance in CD03 (76.1 %), followed by WWTP01–04 (44.3–71.4 %), WWTP05–06 (14.5–15.4 %) and CD01–02 (2.8–9.2 %), while it was not detected at all in CD04. T-RF 396 bp dominated in CD04 (88.8 %) and WWTP05–06 (66.1–80.8 %), while T-RF 78 bp showed higher relative abundance in WWTP06 (13.1 %), WWTP04 (13.7 %) and WWTP03 (23.9 %), but was present at lower levels (<8 %) in the other plants.

Incubation with cellulose and straw in the batch test clearly changed the *cel5* TRFLP profile compared to the one observed at the beginning of the experiment. However, the response varied both with inoculum and with substrate (Fig. 4a). In CD01, the relative abundances of the dominant T-RFs 275 and 362 bp were lower after the incubation with cellulose (28.3, 14.0 %) compared to levels at the beginning of the batch test. Instead the abundance of T-RFs 74, 78, 85, 156 and 396 bp were comparably higher (27.2, 6.0, 11.5, 10.6 and 7.4 %, respectively) than at the starting point (0, 1.8, 2.8, 1.2 and 2.6 %, respectively). For CD02, a similar decrease was seen for T-RF 362 bp, while with this inoculum T-RF 275 bp increased to 79.9 %. The other T-RFs (i.e. 74, 78, 85, 156 and 396 bp) that increased to a smaller extent in CD01 were not detected at the end point for this inoculum. Using straw as substrate resulted in a similar T-RFLP profile change as for cellulose for both CD01 and CD02. For CD03 and WWTP01–04, the relative abundance of the most dominant T-RF 85 bp decreased after incubation with both cellulose and straw. For WWTP03, the level of T-RFs 78 and 85 bp was reduced to non-detectable, while instead T-RFs 275 and 362 bp increased to 76.5 and 23.5 % for cellulose (83.6 and 16.4 % for straw). However, at the end point using both substrates, T-RFs 74, 78 and 156 bp increased their level for CD03, WWTP01–02 and WWTP04, except T-RFs 74 and 156 bp for WWTP04c. For WWTP04c, the level of T-RFs 275 and 362 bp increased. T-RF 122 bp increased in CD03c and WWTP01–02c, while T-RF 130 bp increased in WWTP01–02c. For CD04 and WWTP05–06, the most pronounced change was for the dominant T-RF 396 bp, which decreased to 7.9, 8.1 and 7.8 %, respectively. Instead, for CD04c, T-RFs 211, 85, 156 and 74 bp increased to 44.1, 26.5, 11.9 and 8.2 %, respectively. In addition, for CD04 s T-RF 78 bp increased to 15.8 %. For WWTP05, T-RFs 74, 78, 85 and 275 bp increased to 14.1, 10.5, 37.1 and 12.4 %, respectively, for cellulose, while T-RFs 74, 85 and 275 bp increased to 23.4, 33.7 and 9.5 %, respectively, for straw. The profile of WWTP06 was similar to that of WWTP05 but, in addition, T-RFs 156 and 78 bp increased to 16.1 and 30.5 % for WWTP06c and WWTP06 s, respectively.

#### *cel48*

The T-RFLP profile obtained by the *cel48* primer set of the inoculum samples showed a high relative abundance of T-RF 62 in the majority of samples from the WWTPs, with WWTP01, 02, 03 and 05 having relative abundance of 28.6–81.5 % (Fig. 4b). The level in WWTP04 and WWTP06 was lower, 4.8 and 1.4 %, respectively. This T-RF was only detected in two CDs (CD02–03) and at low levels (1.5–2.3 %). For WWTP04, the dominant T-RFs were instead 141 bp (21.2 %) and 238 bp (38.6 %), while for WWTP06, the two most dominant T-RFs were 357 bp (48.2 %) and 328 bp (12.8 %). For CD01 and CD04, the T-RFLP profile was dominated by T-RFs 357 bp (35.4 and 17.4 %) and 328 bp, (30.1 and 48.0 %). For CD02, the level of T-RF 328 bp was similar (29.9 %), but T-RF 357 bp represented only 1.9 %. Instead, T-RFs 231, 224 and 73 bp were present at a level of 17.9, 11.5 and 10.2 %, respectively. For CD03, the two most dominant T-RFs were 247 bp (34.9 %) and 321 bp (33.0 %), which were present at a relatively low level in all other biogas plants.

In contrast to the *cel5* community, the end point *cel48* community in most cases was not the same when using cellulose and straw as substrate (Fig. 4b). For CD01, the dominant T-RFs 328 and 357 bp decreased in both cases, but T-RF 316 bp (47.9 %) increased in the cellulose incubations and T-RF 296 bp (53.6 %) increased after incubation with straw. For CD02, the dominant T-RF after incubation with straw and cellulose was 268 bp (30.5 %) and 357 bp (61.6 %), respectively. For CD03, the starting point T-RF 247 bp disappeared for both end points, while T-RF 321 bp increased in response to cellulose addition (67.0 %), but declined in abundance when straw was used as substrate (2.5 %). In addition, T-RF 62 bp increased to 89.0 % when straw was used. For CD04, T-RF 357 bp dominated after digestion with cellulose (90.9 %), while T-RFs 321 bp (38.4 %), 328 bp (16.2 %) and 357 bp (14.1 %) dominated in the straw cultures. For WWTPs, after digestion with cellulose, the major peak was T-RF 62 bp (13.0–94.6 %). In addition, T-RFs 296 bp (78.7 %) and 321 bp (45.1 %) were high in WWTP03 and 04, respectively. For the end point with straw, T-RF 62 bp remained as the major peak in WWTP02 (38.9) and 06 (74.1 %) and T-RF 296 bp was also high in WWTP02 (41.4 %). For WWTP01 s and 04–05 s, the dominant peak changed to T-RF 164 bp, with a relative abundance of 94.0, 49.3 and 93.6 %, respectively. For WWTP03 s, T-RF 357 bp, with a level of 36.3 %, was the major peak in this plant.

#### Clone libraries and phylogenetic analysis

Sequencing of 118 and 215 clones from *cel5* and *cel48* libraries resulted in 10 and 11 OTUs, respectively. All OTUs have low similarity to characterised bacteria, with one exception of OTU10, which partial sequence shared 100 % identity to *Clostridium cellulolyticum*. For twenty out of 21 OTUs the closest cultivated bacterial phyla belonged to Bacteroidetes or Firmicutes, one OTU was close to Actinobacteria (*cel48* OTU06, Table 4). This is in agreement with previous studies investigating the bacterial community in biogas digesters using the same primers that all analysed clones were close to Bacteroidetes and Firmicutes [29]. Although the partial deduced amino acid sequences are as short as 100 and 130 amino acid residues, respectively, both trees are supported by high bootstrap values.

**Table 4:** Clone sequences of *cel5* and *cel48* obtained from industrial-scale biogas processes

Clone	T-RFs (bp)	Most closely related microorganism	Identity (%)	Accession number
*Cel5*				
OTU01	56	*Ruminococcus callidus*	84.8	WP_021681794
OTU02^a^	85	*Echinicola vietnamensis*	58.0	WP_015264998
OTU03^b^	85	*Echinicola vietnamensis*	61.4	WP_015264998
OTU04^c^	106	*Flavobacterium* sp.	66.3	WP_007808671
OTU05	136	*Niastella koreensis*	73.7	AEV98714
OTU06^d^	211	*Marinilabilia salmonicolor*	66.3	WP_036163195
OTU07^e^	275	*Mahella australiensis*	57.4	AEE96311
OTU08^f^	362	*Mahella australiensis*	58.4	AEE96311
OTU09	375	*Clostridium* sp.	72.1	WP_033166154
OTU10	396	*Clostridium cellulolyticum*	100	WP_015924614
*Cel48*				
OTU01	68	*Clostridium stercorarium DSM 8532*	57.9	AGI39871
OTU02	68	*Clostridium* sp. *Iso6*-*17a*	50.0	ADM52292
OTU03	177	*Ruminococcus* sp. *CAG:254*	97.5	CCZ84184
OTU04^g^	205	*Clostridium* sp. *Iso6*-*24*	79.0	ADM52293
OTU05	238	*Ruminococcus* sp. *HUN007*	83.2	WP_049962845
OTU06	238	*Streptomyces griseorubens*	61.0	WP_037642616
OTU07^h^	247	*Acetivibrio cellulolyticus*	71.2	WP_010681059
OTU08	290	*Clostridium acetobutylicum*	50.5	WP_010964229
OTU09^i^	321	*Ruminiclostridium thermocellum*	75.0	ACT46162
OTU10^j^	328	*Clostridium termitidis CT1112*	73.8	EMS73539
OTU11^k^	358	*Clostridium straminisolvens JCM 21531*	78.8	GAE90081

^a^Most closely related to uncultured bacterium AGW24153 (identity: 100 %) from laboratory-scale anaerobic reactor

^b^Most closely related to uncultured bacterium AGW24153 (identity: 89.1 %) from laboratory-scale anaerobic reactor

^c^Most closely related to uncultured bacterium ACV50344 (identity: 73.3 %) from lignocellulose-based sulphate-reducing bioreactor

^d^Most closely related to uncultured bacterium AGO64733 (identity: 75.0 %) from anaerobic digester sludge

^e^Most closely related to uncultured bacterium AEV59723 (identity: 77.0 %) from laboratory biogas digester treating rice straw

^f^Most closely related to uncultured bacterium AEV59723 (identity: 76.0 %) from laboratory biogas digester treating rice straw

^g^Most closely related to uncultured bacterium AGO64695 (identity: 99.0 %) from anaerobic digester sludge

^h^Most closely related to uncultured bacterium AGO64692 (identity: 75.2 %) from anaerobic digester sludge

^i^Most closely related to uncultured bacterium AGO64682 (identity: 85.6 %) from anaerobic digester sludge

^j^Most closely related to uncultured bacterium AGO64673 (identity: 99.1 %) from anaerobic digester sludge

^k^Most closely related to uncultured bacterium AGO64695 (identity: 99.0 %) from anaerobic digester sludge

#### *cel5*

**Fig. 5:**
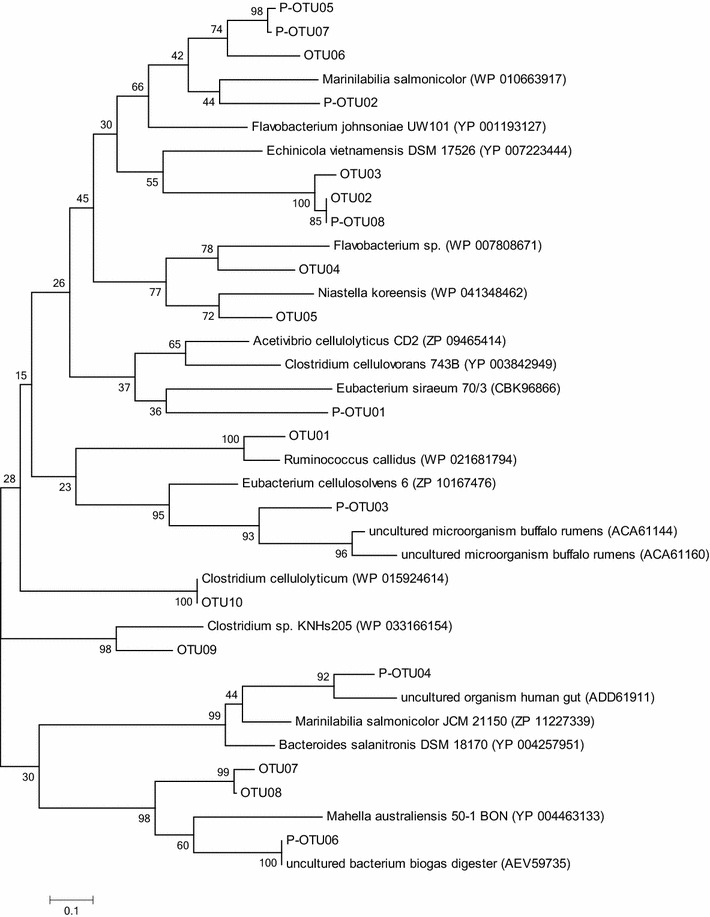
Phylogenetic tree of sequences from glycoside hydrolase gene family 5 retrieved from different industrial-scale biogas processes. *OTUs* operational taxonomic units identified in this study, *P-OTUs* operational taxonomic units identified in Sun et al. [29])

T-RF 275 bp, represented by OTU07 and present in all digesters except CD04, and T-RF 362 bp (OTU08), highly abundant in CD01 and CD02, were both most closely related to an uncultured bacterium (AEV59723), with an identity of 77.0 and 76.0 %, respectively. *Mahella australiensis* (phylum Firmicutes) was the closest cultivated bacterium, with an identity of 57.4 and 58.4 %, respectively (Table 4, Fig. 5). *M. australiensis* is able to ferment different carbohydrates, including cellobiose [78]. It has previously been detected in thermophilic digesters operating with chicken manure, where it is suggested to be sensitive to ammonia [79, 80]. This species has also been observed in the *cel5* community in a CSTR digester fed cow manure and steam-exploded straw operating at 44 °C [29]. T-RF 85 bp, present at high level in most digesters and represented by OTU02 and OTU03, was closely related to an uncultured bacterium (AGW24153), with an identity of 100 and 89.1 %, respectively. This OTU has previously been observed during anaerobic digestion of cow manure and steam-exploded straw at 37 °C [29]. The closest cultivated bacterium was *Echinicola vietnamensis,* with an identity of 58 and 61.4 %, respectively. *E. vietnamensis* was first isolated from seawater [81], and is able to hydrolyse starch and grow up to 44 °C with 15 % NaCl. T-RF 396 bp (OTU10), dominating in CD04, WWTP05 and WWTP06, was verified as *C. cellulolyticum* (identity 100 %). This is a well-studied non-ruminal mesophilic cellulolytic bacterium, initially isolated from decayed grass [82] that has the ability to degrade cellulose and hemicellulose to acetate, ethanol, lactate and H_2_ [83]. Moreover, it has been shown that bioaugmentation with *C. cellulolyticum* can increase the cellulose degradation efficiency of wheat straw during batch cultivation [84]. T-RF 78 bp, which increased in most of the digesters in response to cellulose and straw, was unfortunately not found in the clone library in this study. However, in a previous study investigating the *cel5* community in digesters operating with manure and steam-exploded straw, a T-RF of the same size was shown to correspond to a clone related to *Eubacterium siraeum* (49.5 % identity) [29]. The abundance of the organism in that study was shown to increase from 14  to 52 % with increasing temperature from 37 to 52 °C. This bacterium has been isolated from human faeces and belongs to a genus commonly existing in the human gut and involved in cellulose degradation [85] [86]. T-RF 211 bp, which increased in the CDs in response to both straw and cellulose, was represented by OTU06. This OTU was related to an uncultured bacterium (AGO64733; 75.0 % identity) previously identified in a laboratory-scale reactor operating with manure and straw [29] and grouped with P-OTU05 and 07, identified in the same study (Fig. 5). The closest cultured relative, *Marinilabiliasalmonicolor* (phylum Bacteroidetes, 66.3 % identity) was isolated in seawater and has been shown to have the ability to degrade cellulose, monomeric sugars and starch [87].

#### *cel48*

**Fig. 6:**
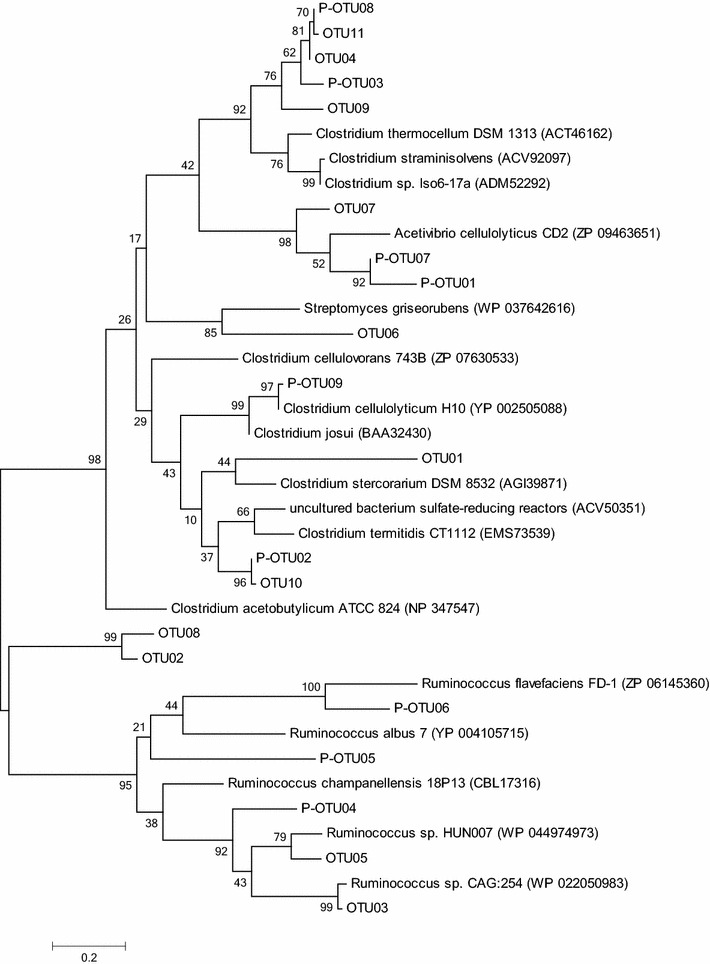
Phylogenetic tree of sequences from glycoside hydrolase gene family 48 retrieved from different industrial-scale biogas processes. *OTUs* operational taxonomic units identified in this study, *P-OTUs* operational taxonomic units identified in Sun et al. [29]

T-RF 238 bp (OTU05), dominating in WWTP04, was most closely related to *Ruminococcus* sp. (identity 82.3 %). T-RFs 358 bp (OTU11) and 328 bp (OTU10), dominating in WWTP06, were most closely related to uncultured bacterium AGO64695 (99.0 % identity) and AGO64673 (99.1 % identity) as identified previously [29]. The closest cultivated relative was *Clostridium straminisolvens* JCM 21,531(78.8 % identity) and *Clostridium termitidis* CT1112 (73.8 % identity) for OTU11 and OTU10, respectively (Fig. 6). Both *C. straminisolvens* and *C. termitidis* have previously been shown to have the ability to degrade a variety of monomeric sugars, as well as cellobiose [88, 89]. *Clostridium straminisolvens* has been detected in various types of biogas digesters, such as an anaerobic thermophilic digester fed municipal waste [24] and an anaerobic mesophilic digester fed pig manure, rice straw [90, 91]. The T-RF with size 238 bp was also found in digester WWTP03 at an abundance of 11.9 %. In this case, the corresponding clone (OTU06) was closely related to *Streptomyces griseorubens* (61 % identity). This result highlights one drawback of T-RFLP analysis, with several different sequences resulting in the same T-RF size, and the importance of combining this method with a clone library. *S. griseorubens* has been isolated from soil in both India and China, and the ability to degrade lignocellulose has been demonstrated [92–94]. Clones OTU07 and OTU09 represented T-RFs 247 bp and 321 bp, respectively, which were present at comparatively high levels in CD03. For these, two uncultured bacteria (AGO64692; identity 75.2 % and AGO64682; identity 85.6 %), respectively, both retrieved from anaerobic digester sludge, had the highest similarity. The closest known bacteria were *Acetivibrio cellulolyticus* (71.2 % identity) and *Ruminiclostridium thermocellum* (75 % identity), respectively*. A. cellulolyticus* can utilise cellulose, cellobiose and salicin and has previously been found in a methanogenic enrichment culture from municipal sewage sludge [95]. *R. thermocellum*, synonym of *Clostridium thermocellum*, is a well-studied anaerobic cellulose-degrading thermophilic organism which has been suggested as a potential candidate for different biotechnological applications [96]. It has been isolated from plants and from cow and horse manure [97] and has been demonstrated to play a key role in cellulolytic degradation in different types of biomethane production digesters [98–100]. After incubation with straw, T-RFs 296 and 164 bp increased in abundance in CD01 and WWTP01, respectively. These two T-RFs could not be found in the clone libraries. However, in a previous study of laboratory-scale digesters operating with manure and straw at 44 °C [29], both T-RFs were found and suggested to represent species closely related to *A. cellulolyticum* (74.3 % identity) and *Ruminococcus champanellensis* (61.8 % identity), respectively. *R. champanellensis* is able to utilise cellulose, cellobiose and xylan, but not starch and pectin [101]. OTU02 (T-RF 68) and OTU08 (T-RF 290) grouped together in the phylogenetic tree (Fig. 6) and showed similarity to *Ruminococcus*, but with relatively low identity (50.0 and 50.5 %, respectively). OTU04, 09 and 11 grouped together with P-OTU03 and 08 from a previous study investigating potential cellulose-degrading bacteria in digesters operating with straw and cow manure [29].

### Correlation of microbial community structure with process parameters

**Fig. 7:**
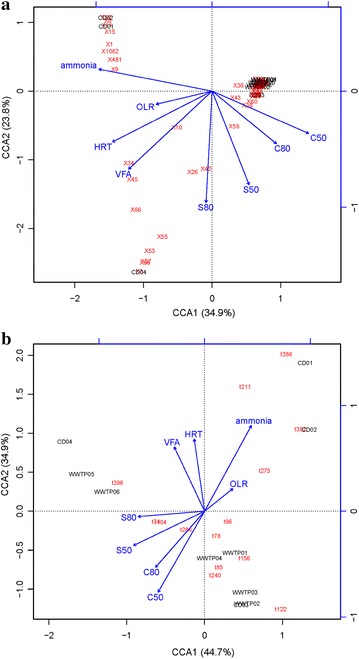
Canonical correspondence analysis (CCA) of **a** the major OTUs at genus level of the 16S rRNA gene and **b** the T-RFs of *cel5* community, within 10 industrial-scale biogas plants. *CD 01–04* co-digestion plants, *WWTP 01–06* wastewater treatment plants. *Ammonia* free ammonia, *OLR* organic loading rate, *HRT* hydraulic retention time, *VFA* volatile fatty acids, C50/80 and S50/80: the inverse of days needed to reach 50/80 % of final methane potential for cellulose and straw, respectively

To identify possible correlations between microbial community composition within the inoculum and biogas process operating parameters (Table 1) and batch digestion performance (Table 2) using cellulose and straw as substrate, CCA was performed. On including the process parameters (free ammonia, OLR, HRT and VFA) and process performance (inverse of days needed to reach 50 and 80 % of the final methane potential for cellulose and straw, C50/80 and S50/80), the first two dimensions of the CCA plot explained around 58.7 % (16S amplicon sequencing; Fig. 7a) and 79.6 % (*cel5*, Fig. 7b) of the variation in relative abundance of OTUs and T-RFs, respectively. For *cel48*, no clear clustering of community structure or clear influence of parameter was found (data not shown).

The CCA plot of amplicon sequencing data (Fig. 7a) showed a similar pattern to the weighted UniFrac PCoA plot (Fig. 2b). The free ammonia concentration was identified as the one important factor influencing the community structure, determined by analysis of both the 16S rRNA and cel5 gene. Batch degradation performance, i.e. the time to reach 50 and 80 % degradation of both cellulose and straw, on the other hand, was negatively associated with the level of free ammonia in the original process in both analyses. At present, not much information is available in the literature regarding the impact of ammonia on the degradation of cellulose and the obtained results so far are contradictory [21]. In the 16S rRNA amplicon dataset, a few OTUs were found to be positively correlated with ammonia level, including the highly dominant OTU in both digester CD01 and CD02. This OTU was also negatively correlated with the batch process performance. For the cel5 community, the dominant T-RFs 362 bp showed a positive correlation with ammonia concentration, while the T-RFs 396 and 85 bp were correlated with process performance. One of these T-RFs (TRF 396) represented a clone with 100 % similarity to *C. cellulolyticum*, recently shown to increase the cellulose degradation efficiency of wheat straw during bioaugmentation [84]. The batch test results cannot be completely transferred to the industrial-scale process, i.e. the degradation in the batch test might not be the same as in the continuous full-scale system. Still the microbial composition in the inoculums, shaped by the process parameters in the full-scale plant, will most likely impact on the outcome of the BMP test, as has been shown in previous studies [102].

## Conclusions

The overall bacterial communities within the investigated co-digestion and WWTP plants were separated from each other, probably owing to differences in substrate and/or operating parameters used by these two groups of biogas plants. Moreover, the diversity was lower in CD compared with WWTP plants. Among the ten plants, two showed clearly lower degradation efficiency of straw and cellulose, measured during batch cultivation. These two plants also had the lowest bacterial diversity (species richness and evenness) and the highest level of ammonia. Ten of 21 OTUs obtained from clone libraries based on the glycosidase hydrolase gene sequence were mainly distantly related to known organisms, while the rest were related to partial sequences of unknown bacteria, although according to the phylogenetic analysis still related to saccharolytic or cellulolytic bacteria. Statistical analysis identified ammonia as a parameter with a strong impact on the *cel5* community, while no clear trend could be seen for the *cel48* community. This indicates that ammonia not only influences the methanogenic community structure in biogas processes, but also shapes the community of bacteria involved in the hydrolysis step. Interestingly, two dominant T-RFs from the *cel5* community were positively correlated with batch process performance, i.e. the degradation efficiency of straw and cellulose. One of these T-RFs represented a clone with 100 % similarity to *C. cellulolyticum*, recently shown to be of importance for the degradation of wheat straw. The presence of this bacterium was negatively correlated to the ammonia level, supporting the idea that ammonia might have a negative impact on the degradation of lignocellulosic material.

## Methods

### Biogas plants

In total, the main digesters of 10 different industrial-scale biogas plants were investigated. These digesters were all operating at mesophilic temperature (range 37–38 °C), six processing sludge from wastewater treatment plants (WWTP01–06) and four co-digesting various organic waste fractions (CD01–04), such as agricultural waste, source-separated organic municipal household waste and slaughterhouse waste (Table 1). All investigated biogas plants had been in operation for several years under similar conditions regarding substrate and operating parameters.

### Bio-methane potential test

The methane potential of cellulose and straw was determined by biochemical methane potential (BMP) analysis [103]. The substrate used in the BMP test was cellulose (C6663, Sigma-Aldrich, MO, USA) and wheat straw (mechanical chopped into 1–2 cm). Inoculum was collected from the different biogas plants and incubated at 37 °C for 4–6 days prior to the BMP test to decrease gas production from endogenous material. The batch anaerobic digestion was performed in 309 mL serum bottles, to which substrate was added corresponding to 3 g volatile solids (VS) per liter, and the inoculum:substrate ratio was between 2:1 and 4:1. The final liquid volume was set to 193 mL in all bottles by adding tap water. For each inoculum, triplicate bottles were set up for straw and for cellulose. Triplicate bottles filled only with the inoculum, corresponding to the same volume as in the test bottles, were also set up to measure background production of methane (CH_4_). All bottles were incubated at 37 °C on a rotary shaker at 130 rpm. Total gas production and methane content were continuously monitored over 60 days by pressure measurement combined with gas sampling and gas chromatograph (GC) analysis of gas composition [103]. The accumulated methane production was calculated and the gas production from the control was deducted. The volumetric methane value was normalised to standard temperature (273.15 K) and pressure (1 bar) using the ideal gas law, and finally expressed as N mL CH_4_ g VS^−1^ [104]. The days needed to reach 50 and 80 % of final methane potential was used as a measure of degradation capacity.

### Analytical methods

Content of total solids (TS) and VS in the inoculum samples was measured according to the international standard methods [105]. Total Kjeldahl nitrogen (TKN) and ammonium-nitrogen (NH_4_-N) were analysed according to the International Standardization Operation (ISO) methods (ISO 10,694, 1995 and ISO 13,878, 1998). The concentration of free ammonia was calculated from the NH_4_-N concentration, pH and temperature according to Hansen et al. [106]. The volatile fatty acid (VFA) content in the digester samples was determined by high-performance liquid chromatography (HPLC) analysis as described previously [103].

### DNA extraction

Samples for microbial community analysis were taken from the inoculum on the start day of the BMP test and at the end of the anaerobic batch test. A 15 mL sample was withdrawn from each inoculum and each batch test. The latter samples were designated CD 01–04c, WWTP 01–06c and CD 01–04 s, WWTP 01–06 s, for samples from incubation with cellulose and straw, respectively. All samples were stored at −20 °C until the extraction of DNA. Total genomic DNA was extracted in triplicate using the FastDNA Spin kit for soil (MP Biomedicals, Santa Ana, CA, USA) according to the manufacturer’s instructions, with the modifications that aliquots of 200 μL digester sample were used for extraction and 60 μL water was used in the final DNA elution. The concentrations of extracted DNA were measured using a Nano Vue spectrophotometer (GE Healthcare, Buckinghamshire, UK).

### 454-pyrosequencing and 16S rRNA gene sequence analysis

The bacterial communities in inoculum from the 10 industrial biogas plants were investigated by amplification of genomic DNA using polymerase chain reaction (PCR) primers targeting the bacterial 16S rRNA gene and integrated with 454 Life Sciences adaptors 8F (5′-CCT ATC CCC TGT GTG CCT TGG CAG TCT CAG CAA CAG CTA GAG TTT GAT CCT GG-3′) and 515R (5′-CCA TCT CAT CCC TGC GTG TCT CCG ACT CAG NNN NNN NNT TAC CGC GGC TGC T-3′ [107]. Each PCR contained 12.5 µL of Maxima Hot Start PCR Master Mix (Fermentas, Thermo Fisher Scientific, Hudson, NH, USA), 0.5 µM of each primer, 20 ng of DNA template and 9.5 µL of sterile water (25 µL final volume). The PCR protocol was as described in Sun et al. [31]. The size and purity of amplicons were checked by electrophoresis on 2 % agarose gel. Short, non-specific amplification products were removed with AMP beads (AMPure XP, Beckman Coulter Genomics, Danvers, MA, USA) using the manufacturer’s protocol but with a modified bead: DNA volume ratio of 0.7:1. The concentrations of purified products were measured using the Quant-iT dsDNA BR Assay Kit (Invitrogen, Life Technologies Europe, Stockholm, Sweden). All PCR products were pooled in equal molar amounts and sequenced at the Swedish Institute for Infectious Disease Control in Solna (Stockholm, Sweden), using the Roche/454 GS Titanium technology platform. The 16S rRNA sequences were processed as described previously [31] and deposited in the NCBI Sequence Read Archive (SRA) under the accession number PRJNA290173.

### T-RFLP

Primers targeting the glycoside hydrolase families 5 (cel5_392F 5′-GAG CAT GGG CTG GAA YHT NGG NAA-3′ and cel5_754R 5′-CAT CAT AAT CTT TGA AGT GGT TTG CAA TYT GDK TCC A-3′) and 48 (cel48_490F 5′ TNA TGG TTG AAG CTC CDG AYT AYG G-3′ and cel48_920R 5′-CCA AAN CCR TAC CAG TTR TCA ACR TC-3′) [86] were used to study the cellulose-degrading bacterial community structures in the different inoculum samples and at the end of the batch test by terminal restriction fragment length polymorphism (T-RFLP) analysis. For the assay, the 5′end of the cel5_754R and cel48_920R primer was labelled with 6-carboxyfluorescein (FAM). PCR amplification of triplicate extractions was conducted using Maxima Hot Start PCR Master Mix (Fermentas, Thermo Fisher Scientific, Hudson, NH, USA). Each PCR contained 12.5 µL of corresponding reaction mix, 1 µL of each primer (0.5 µM final concentration), 1 µL of DNA template (20 times dilution) and 9.5 µL of sterile water. The PCR program used for amplification of *cel5* and *cel48* included initialisation at 95 °C for 5 min, denaturation at 95 °C for 1 min, annealing for 30 s at 56 °C for *cel48* (35 cycles) or at 52 °C for *cel5* (45 cycles), elongation at 72 °C for 30 s, followed by a final extension at 72 °C for 10 min. The pooled FAM-labelled amplicons of *cel5* and *cel48* were purified with QIAquick gel extraction kit (Qiagen, Hilden, Germany) and digested overnight at 37 °C with restriction enzyme *Mbo*I (New England Biolabs, Wilbury Way Hitchin, Herts, UK) and *Alu*I (Fermentas, Thermo Fisher Scientific, Hudson, NH, USA). Fluorescently labelled terminal restriction fragments (T-RFs) were separated and detected with ABI3730xl capillary sequencer (Applied Biosystems, Cheshire, UK). GS ROX 500 internal size standard (Applied Biosystems) was included in all assays. The T-RFLP profiles were processed by Peak Scanner software (1.0, Applied Biosystems) and the relative abundance of the individual T-RFs was calculated by dividing the peak area by the total area of all peaks. T-RFs shorter than 70 bp (*cel5*) and 50 bp (*cel48*) constituting less than 1 % of the total peak area were excluded as background.

### Clone library construction and sequencing analysis

Clone libraries were constructed for the *cel5* community with samples retrieved from CD01 and WWTP 02c/03/04/05c and for the *cel48* community with samples from CD 01/02/03 and WWTP 03/04/06, as described previously [29]. In brief, triplicate PCRs were conducted for each DNA extraction replicate using the primer (without FAM label) and conditions described above. The resulting nine PCR products per sample were pooled and gel purified with QIAquick gel extraction kit (Qiagen, Hilden, Germany) and ligated into pCR™4-TOPO^®^ vector (Invitrogen, Life Technologies, Grand Island, NY, USA), followed by transformation of the ligation product into TOP10 One Shot^®^ chemically competent *Escherichia coli* (Invitrogen), according to the manufacturer’s instructions. The sequences obtained were quality checked and edited with the software package Geneious, version 5.6.5 (Biomatters Ltd., Auckland, New Zealand) and subsequently assigned to operational taxonomic units (OTU) at the threshold of 97 % nucleotide identity. The sequences were compared with sequences available in the NCBI GenBank. Alignment of cloned sequences and selected reference sequences, as well as sequences from uncultured bacteria, was conducted using the programme MUSCLE [108]. The phylogenetic trees were constructed with the MEGA programme version 5 using the maximum likelihood method and WAG model [109]. The confidence of the trees was tested by bootstrap resampling analysis for 1000 replicates. All sequences were deposited in the NCBI GenBank database under the accession number KT336110-29 for primer pair *cel5* and KT336130-99 for primer *cel48*.

### Statistics

To investigate correlations between the microbial composition of the different biogas plants and the batch test performance using inoculum from the corresponding plants, canonical correspondence analysis (CCA) was performed using the Vegan Community Ecology Package (version 2.3-0, http://CRAN.R-project.org/package=vegan) within R (A language and environment for statistical computing, http://www.R-project.org/). Separate CCA was performed using the amplicon sequencing and T-RFLP data. Only major OTUs, i.e. OTUs representing ≥2 % of total sequences at genus level, were selected for the assay and for T-RFLP the relative abundance of each T-RF was used. The inverse of days needed to reach 50 and 80 % of final methane potential was used as a measure of degradation performance. The process data (Table 1) were included as environmental variables.

## Additional file

10.1186/s13068-016-0543-9 **Figure S1** Relative abundance of bacterial 16S rRNA gene at class level in 10 industrial-scale biogas plants. CD 01-04: co-digestion plants; WWTP 01-06: wastewater treatment plants: **Figure S2**. Relative abundance of bacterial 16S rRNA gene at order level in 10 industrial-scale biogas plants. CD 01-04: co-digestion plants; WWTP 01-06: wastewater treatment plants. **Figure S3**. Relative abundance of bacterial 16S rRNA gene at family level in 10 industrial-scale biogas plants. CD 01-04: co-digestion plants; WWTP 01-06: wastewater treatment plants. **Figure S4**. Relative abundance of bacterial 16S rRNA gene at genus level in 10 industrial-scale biogas plants. CD 01-04: co-digestion plants; WWTP 01-06: wastewater treatment plants. **Figure S5**. OTU heatmap based on bacterial OTUs having relative abundance higher or equal to 0.2% in 10 industrial-scale biogas plants. CD 01-04: co-digestion plants; WWTP 01-06: wastewater treatment plants.

## Abbreviations

AD: anaerobic digestion
BMP: biochemical methane potential test
CCA: canonical correspondence analysis
CD: co-digestion
*cel5*: glycoside hydrolase genes of family 5
*cel48*: glycoside hydrolase genes of family 48
FAM: phosphoramidite fluorochrome 5-carboxy-fluorescein
GC: gas chromatograph
HPLC: high pressure liquid chromatography
HRT: hydraulic retention time
OLR: organic loading rate
OTU: operational taxonomic unit
PCoA: principal coordinates analysis
PCR: polymeras chain reaction
TKN: total kjeldahl nitrogen
TRFLP: terminal restriction fragment length polymorphism
T-RF: terminal restriction fragment
TS: total solids
VFA: volatile fatty acids
VS: volatile solids
WWTP: waste water treatment plant
