# Supplementary material for: The microbial community structure in industrial biogas plants influences the degradation rate of straw and cellulose in batch tests
Source: Biotechnol Biofuels. 2016 Jun 18;9:128. doi: 10.1186/s13068-016-0543-9 (PMC4912747; doi:10.1186/s13068-016-0543-9)
Supplement: Supplementary file 1 — 10.1186/s13068-016-0543-9 Figure S1 Relative abundance of bacterial 16S rRNA gene at class level in 10 industrial-scale biogas plants. CD 01-04: co-digestion plants; WWTP 01-06: wastewater treatment plants: Figure S2. Relative abundance of bacterial 16S rRNA gene at order level in 10 industrial-scale biogas plants. CD 01-04: co-digestion plants; WWTP 01-06: wastewater treatment plants. Figure S3. Relative abundance of bacterial 16S rRNA gene at family level in 10 industrial-scale biogas plants. CD 01-04: co-digestion plants; WWTP 01-06: wastewater treatment plants. Figure S4. Relative abundance of bacterial 16S rRNA gene at genus level in 10 industrial-scale biogas plants. CD 01-04: co-digestion plants; WWTP 01-06: wastewater treatment plants. Figure S5. OTU heatmap based on bacterial OTUs having relative abundance higher or equal to 0.2% in 10 industrial-scale biogas plants. CD 01-04: co-digestion plants; WWTP 01-06: wastewater treatment plants. [file 13068_2016_543_MOESM1_ESM.docx]

Figure S1.

Figure S2.

Figure S3.

Figure S4.

Figure S5.

| OTU ID | CD01 | CD02 | CD04 | CD03 | WWTP01 | WWTP02 | WWTP03 | WWTP04 | WWTP05 | WWTP06 | **Consensus Lineage** |
| --- | --- | --- | --- | --- | --- | --- | --- | --- | --- | --- | --- |
| 1 |  |  |  |  |  |  |  |  |  |  | k__Bacteria |
| 50 |  |  |  |  |  |  |  |  |  |  | k__Bacteria;p__Actinobacteria;c__Actinobacteria;o__Actinomycetales;f__Actinomycetaceae;g__Actinomyces;s__europaeus |
| 61 |  |  |  |  |  |  |  |  |  |  | k__Bacteria;p__Actinobacteria;c__Thermoleophilia;o__Gaiellales;f__;g__;s__ |
| 9 |  |  |  |  |  |  |  |  |  |  | k__Bacteria;p__Bacteroidetes;c__Bacteroidia;o__Bacteroidales |
| 13 |  |  |  |  |  |  |  |  |  |  | k__Bacteria;p__Bacteroidetes;c__Bacteroidia;o__Bacteroidales |
| 53 |  |  |  |  |  |  |  |  |  |  | k__Bacteria;p__Bacteroidetes;c__Bacteroidia;o__Bacteroidales |
| 1082 |  |  |  |  |  |  |  |  |  |  | k__Bacteria;p__Bacteroidetes;c__Bacteroidia;o__Bacteroidales |
| 5 |  |  |  |  |  |  |  |  |  |  | k__Bacteria;p__Bacteroidetes;c__Bacteroidia;o__Bacteroidales;f__;g__;s__ |
| 6 |  |  |  |  |  |  |  |  |  |  | k__Bacteria;p__Bacteroidetes;c__Bacteroidia;o__Bacteroidales;f__;g__;s__ |
| 8 |  |  |  |  |  |  |  |  |  |  | k__Bacteria;p__Bacteroidetes;c__Bacteroidia;o__Bacteroidales;f__;g__;s__ |
| 30 |  |  |  |  |  |  |  |  |  |  | k__Bacteria;p__Bacteroidetes;c__Bacteroidia;o__Bacteroidales;f__;g__;s__ |
| 38 |  |  |  |  |  |  |  |  |  |  | k__Bacteria;p__Bacteroidetes;c__Bacteroidia;o__Bacteroidales;f__;g__;s__ |
| 42 |  |  |  |  |  |  |  |  |  |  | k__Bacteria;p__Bacteroidetes;c__Bacteroidia;o__Bacteroidales;f__;g__;s__ |
| 58 |  |  |  |  |  |  |  |  |  |  | k__Bacteria;p__Bacteroidetes;c__Bacteroidia;o__Bacteroidales;f__;g__;s__ |
| 617 |  |  |  |  |  |  |  |  |  |  | k__Bacteria;p__Bacteroidetes;c__Bacteroidia;o__Bacteroidales;f__;g__;s__ |
| 26 |  |  |  |  |  |  |  |  |  |  | k__Bacteria;p__Bacteroidetes;c__Bacteroidia;o__Bacteroidales;f__Bacteroidaceae;g__;s__ |
| 43 |  |  |  |  |  |  |  |  |  |  | k__Bacteria;p__Bacteroidetes;c__Bacteroidia;o__Bacteroidales;f__Bacteroidaceae;g__;s__ |
| 15 |  |  |  |  |  |  |  |  |  |  | k__Bacteria;p__Bacteroidetes;c__Bacteroidia;o__Bacteroidales;f__Porphyromonadaceae;g__;s__ |
| 18 |  |  |  |  |  |  |  |  |  |  | k__Bacteria;p__Bacteroidetes;c__Bacteroidia;o__Bacteroidales;f__Porphyromonadaceae;g__;s__ |
| 0 |  |  |  |  |  |  |  |  |  |  | k__Bacteria;p__Bacteroidetes;c__Bacteroidia;o__Bacteroidales;f__SB-1;g__;s__ |
| 413 |  |  |  |  |  |  |  |  |  |  | k__Bacteria;p__Bacteroidetes;c__Bacteroidia;o__Bacteroidales;f__SB-1;g__;s__ |
| 1107 |  |  |  |  |  |  |  |  |  |  | k__Bacteria;p__Bacteroidetes;c__Bacteroidia;o__Bacteroidales;f__SB-1;g__;s__ |
| 44 |  |  |  |  |  |  |  |  |  |  | k__Bacteria;p__Chloroflexi;c__Anaerolineae;o__Anaerolineales;f__Anaerolinaceae;g__;s__ |
| 14 |  |  |  |  |  |  |  |  |  |  | k__Bacteria;p__Chloroflexi;c__Anaerolineae;o__Anaerolineales;f__Anaerolinaceae;g__T78;s__ |
| 16 |  |  |  |  |  |  |  |  |  |  | k__Bacteria;p__Chloroflexi;c__Anaerolineae;o__Anaerolineales;f__Anaerolinaceae;g__T78;s__ |
| 20 |  |  |  |  |  |  |  |  |  |  | k__Bacteria;p__Chloroflexi;c__Anaerolineae;o__Anaerolineales;f__Anaerolinaceae;g__T78;s__ |
| 32 |  |  |  |  |  |  |  |  |  |  | k__Bacteria;p__Chloroflexi;c__Anaerolineae;o__Anaerolineales;f__Anaerolinaceae;g__T78;s__ |
| 36 |  |  |  |  |  |  |  |  |  |  | k__Bacteria;p__Chloroflexi;c__Anaerolineae;o__Anaerolineales;f__Anaerolinaceae;g__T78;s__ |
| 64 |  |  |  |  |  |  |  |  |  |  | k__Bacteria;p__Chloroflexi;c__Anaerolineae;o__Anaerolineales;f__Anaerolinaceae;g__T78;s__ |
| 321 |  |  |  |  |  |  |  |  |  |  | k__Bacteria;p__Chloroflexi;c__Anaerolineae;o__Anaerolineales;f__Anaerolinaceae;g__T78;s__ |
| 51 |  |  |  |  |  |  |  |  |  |  | k__Bacteria;p__Chloroflexi;c__Anaerolineae;o__Anaerolineales;f__Anaerolinaceae;g__WCHB1-05;s__ |
| 19 |  |  |  |  |  |  |  |  |  |  | k__Bacteria;p__Firmicutes |
| 46 |  |  |  |  |  |  |  |  |  |  | k__Bacteria;p__Firmicutes;c__Bacilli;o__Turicibacterales;f__Turicibacteraceae;g__Turicibacter;s__ |
| 10 |  |  |  |  |  |  |  |  |  |  | k__Bacteria;p__Firmicutes;c__Clostridia;o__Clostridiales |
| 25 |  |  |  |  |  |  |  |  |  |  | k__Bacteria;p__Firmicutes;c__Clostridia;o__Clostridiales |
| 28 |  |  |  |  |  |  |  |  |  |  | k__Bacteria;p__Firmicutes;c__Clostridia;o__Clostridiales |
| 31 |  |  |  |  |  |  |  |  |  |  | k__Bacteria;p__Firmicutes;c__Clostridia;o__Clostridiales |
| 55 |  |  |  |  |  |  |  |  |  |  | k__Bacteria;p__Firmicutes;c__Clostridia;o__Clostridiales |
| 54 |  |  |  |  |  |  |  |  |  |  | k__Bacteria;p__Firmicutes;c__Clostridia;o__Clostridiales;f__;g__;s__ |
| 3 |  |  |  |  |  |  |  |  |  |  | k__Bacteria;p__Firmicutes;c__Clostridia;o__Clostridiales;f__[Tissierellaceae];g__Gallicola;s__ |
| 22 |  |  |  |  |  |  |  |  |  |  | k__Bacteria;p__Firmicutes;c__Clostridia;o__Clostridiales;f__[Tissierellaceae];g__Sedimentibacter;s__ |
| 39 |  |  |  |  |  |  |  |  |  |  | k__Bacteria;p__Firmicutes;c__Clostridia;o__Clostridiales;f__[Tissierellaceae];g__Sedimentibacter;s__ |
| 481 |  |  |  |  |  |  |  |  |  |  | k__Bacteria;p__Firmicutes;c__Clostridia;o__Clostridiales;f__Caldicoprobacteraceae;g__Caldicoprobacter;s__ |
| 67 |  |  |  |  |  |  |  |  |  |  | k__Bacteria;p__Firmicutes;c__Clostridia;o__Clostridiales;f__Clostridiaceae;g__02d06;s__ |
| 12 |  |  |  |  |  |  |  |  |  |  | k__Bacteria;p__Firmicutes;c__Clostridia;o__Clostridiales;f__Clostridiaceae;g__SMB53;s__ |
| 40 |  |  |  |  |  |  |  |  |  |  | k__Bacteria;p__Firmicutes;c__Clostridia;o__Clostridiales;f__Lachnospiraceae;g__Butyrivibrio;s__ |
| 59 |  |  |  |  |  |  |  |  |  |  | k__Bacteria;p__Firmicutes;c__Clostridia;o__Clostridiales;f__Peptococcaceae;g__Pelotomaculum;s__ |
| 34 |  |  |  |  |  |  |  |  |  |  | k__Bacteria;p__Firmicutes;c__Clostridia;o__MBA08;f__;g__;s__ |
| 45 |  |  |  |  |  |  |  |  |  |  | k__Bacteria;p__Firmicutes;c__Clostridia;o__MBA08;f__;g__;s__ |
| 66 |  |  |  |  |  |  |  |  |  |  | k__Bacteria;p__Firmicutes;c__Clostridia;o__MBA08;f__;g__;s__ |
| 11 |  |  |  |  |  |  |  |  |  |  | k__Bacteria;p__Firmicutes;c__Clostridia;o__SHA-98;f__;g__;s__ |
| 35 |  |  |  |  |  |  |  |  |  |  | k__Bacteria;p__Firmicutes;c__Clostridia;o__SHA-98;f__;g__;s__ |
| 2 |  |  |  |  |  |  |  |  |  |  | k__Bacteria;p__Hyd24-12;c__;o__;f__;g__;s__ |
| 27 |  |  |  |  |  |  |  |  |  |  | k__Bacteria;p__OD1;c__;o__;f__;g__;s__ |
| 69 |  |  |  |  |  |  |  |  |  |  | k__Bacteria;p__OP8;c__;o__;f__;g__;s__ |
| 4 |  |  |  |  |  |  |  |  |  |  | k__Bacteria;p__OP8;c__OP8_1;o__;f__;g__;s__ |
| 41 |  |  |  |  |  |  |  |  |  |  | k__Bacteria;p__Proteobacteria;c__Deltaproteobacteria;o__Syntrophobacterales;f__Syntrophaceae;g__Syntrophus;s__ |
| 49 |  |  |  |  |  |  |  |  |  |  | k__Bacteria;p__Proteobacteria;c__Deltaproteobacteria;o__Syntrophobacterales;f__Syntrophaceae;g__Syntrophus;s__ |
| 47 |  |  |  |  |  |  |  |  |  |  | k__Bacteria;p__Proteobacteria;c__Deltaproteobacteria;o__Syntrophobacterales;f__Syntrophorhabdaceae;g__;s__ |
| 17 |  |  |  |  |  |  |  |  |  |  | k__Bacteria;p__SAR406;c__AB16;o__noFP_H7;f__;g__;s__ |
| 21 |  |  |  |  |  |  |  |  |  |  | k__Bacteria;p__Spirochaetes;c__Spirochaetes;o__Spirochaetales;f__Spirochaetaceae;g__Treponema;s__ |
| 29 |  |  |  |  |  |  |  |  |  |  | k__Bacteria;p__Synergistetes;c__Synergistia;o__Synergistales;f__Thermovirgaceae;g__;s__ |
| 60 |  |  |  |  |  |  |  |  |  |  | k__Bacteria;p__Tenericutes;c__Mollicutes;o__Acholeplasmatales;f__Acholeplasmataceae;g__Acholeplasma;s__ |
| 24 |  |  |  |  |  |  |  |  |  |  | k__Bacteria;p__Verrucomicrobia;c__Verruco-5;o__LD1-PB3;f__;g__;s__ |
| 23 |  |  |  |  |  |  |  |  |  |  | k__Bacteria;p__Cloacimonetes;c__[Cloacamonae];o__[Cloacamonales];f__[Cloacamonaceae] |
| 7 |  |  |  |  |  |  |  |  |  |  | k__Bacteria;p__Cloacimonetes;c__[Cloacamonae];o__[Cloacamonales];f__[Cloacamonaceae];g__BHB21;s__ |
| 377 |  |  |  |  |  |  |  |  |  |  | k__Bacteria;p__Cloacimonetes;c__[Cloacamonae];o__[Cloacamonales];f__[Cloacamonaceae];g__BHB21;s__ |
| 37 |  |  |  |  |  |  |  |  |  |  | k__Bacteria;p__Cloacimonetes;c__[Cloacamonae];o__[Cloacamonales];f__[Cloacamonaceae];g__Candidatus Cloacamonas;s__ |
| 52 |  |  |  |  |  |  |  |  |  |  | k__Bacteria;p__Cloacimonetes;c__[Cloacamonae];o__[Cloacamonales];f__[Cloacamonaceae];g__Candidatus Cloacamonas;s__ |
| 63 |  |  |  |  |  |  |  |  |  |  | k__Bacteria;p__Cloacimonetes;c__[Cloacamonae];o__[Cloacamonales];f__[Cloacamonaceae];g__W22;s__ |
| 57 |  |  |  |  |  |  |  |  |  |  | k__Bacteria;p__Cloacimonetes;c__[Cloacamonae];o__[Cloacamonales];f__[Cloacamonaceae];g__W5;s__ |

|  |  |  |  |  |
| --- | --- | --- | --- | --- |
|  |  |  |  |  |
| 0 | |  | 3.26 | |
|  |  | | |  |
| Log abundance of OTUs | | | | |
|  |  |  |  |  |
|  |  |  |  |  |
